# Short- and long-term outcomes of Roux-en-Y and Billroth II with Braun reconstruction in total laparoscopic distal gastrectomy: a retrospective analysis

**DOI:** 10.1186/s12957-023-03249-6

**Published:** 2023-11-22

**Authors:** Yan-xin Chen, Qiao-zhen Huang, Peng-cheng Wang, Yue-Jia Zhu, Li-quan Chen, Chu-ying Wu, Jin-tian Wang, Jun-xing Chen, Kai Ye

**Affiliations:** Department of Gastrointestinal Surgery, The Second Affiliated Hospital of Fujian Medical University, No. 950 Donghai Street, Fengze District, Quanzhou, 362000 Fujian China

**Keywords:** Roux-en-Y, Billroth II with Braun, Bile reflux, Long-term outcomes, Quality of life, Total laparoscopic distal gastrectomy

## Abstract

**Background:**

The controversy surrounding Roux-en-Y (R-Y) and Billroth II with Braun (BII + B) reconstruction as an anti-bile reflux procedure after distal gastrectomy has persisted. Recent studies have demonstrated their efficacy, but the long-term outcomes and postoperative quality of life (QoL) among patients have yet to be evaluated. Therefore, we compared the short-term and long-term outcomes of the two procedures as well as QoL.

**Methods:**

The clinical data of 151 patients who underwent total laparoscopic distal gastrectomy (TLDG) at the Gastrointestinal Surgery Department of the Second Hospital of Fujian Medical University from January 2016 to December 2019 were retrospectively analyzed. Of these, 57 cases with Roux-en-Y procedure (R-Y group) and 94 cases with Billroth II with Braun procedure were included (BII + B group). Operative and postoperative conditions, early and late complications, endoscopic outcomes at year 1 and year 3 after surgery, nutritional indicators, and quality of life scores at year 3 postoperatively were compared between the two groups.

**Results:**

The R-Y group recorded a significantly longer operative time (194.65 ± 21.52 vs. 183.88 ± 18.02 min) and anastomotic time (36.96 ± 2.43 vs. 27.97 ± 3.74 min) compared to the BII + B group (*p* < 0.05). However, no other significant differences were observed in terms of perioperative variables, including blood loss (*p* > 0.05). Both groups showed comparable rates of early and late complications. Endoscopic findings indicated similar food residuals at years 1 and 3 post-surgery for both groups. The R-Y group had a lower occurrence of residual gastritis and bile reflux at year 1 and year 3 after surgery, with a statistically significant difference (*p* < 0.001). Reflux esophagitis was not significantly different between the R-Y and BII + B groups in year 1 after surgery (*p* = 0.820), but the R-Y group had a lower incidence than the BII + B group in year 3 after surgery (*p* = 0.023). Nutritional outcomes at 3 years after surgery did not differ significantly between the two groups (*p* > 0.05). Quality of life scores measured by the QLQ-C30 scale were not significantly different between the two groups. However, on the QLQ-STO22 scale, the reflux score was significantly lower in the R-Y group than in the BII + B group (0 [0, 0] vs. 5.56 [0, 11.11]) (*p* = 0.003). The rest of the scores were not significantly different (*p* > 0.05).

**Conclusion:**

Both R-Y and B II + B reconstructions are equally safe and efficient for TLDG. Nevertheless, the R-Y reconstruction reduces the incidence of residual gastritis, bile reflux, and reflux esophagitis, as well as postoperative reflux symptoms, and provides a better quality of life for patients. R-Y reconstruction is superior to BII + B reconstruction for TLDG.

## Introduction

Quality of life (QoL) among gastric cancer patients post-surgery has become a major focus for surgeons [1]. Changes in digestive tract continuity often result in complications following distal gastrectomy, such as alkaline reflux gastritis, dumping syndrome, and delayed gastric emptying [2]. Among these complications, heartburn and abdominal pain caused by alkaline reflux gastritis heavily impact patient QoL [3]. Previously, the Billroth II procedure was reportedly accompanied by significant bile reflux [4]. The Roux-en-Y (R-Y) method has a relatively better long-term prognosis due to the superior anti-reflux effect [5]. Consequently, surgeons perform Braun anastomosis to reduce bile reflux after Billroth II. Nonetheless, earlier studies exhibited unsatisfactory outcomes [6].

Since the development and widespread use of laparoscopic distal gastrectomy (LDG), the oncologic safety and surgical security have been proven comparable to those of open surgery [7]. Currently, laparoscopic-assisted distal gastrectomy (LADG) remains the primary approach for LDG. However, with the advancement of laparoscopic techniques and linear staplers, attention has shifted toward total laparoscopic distal gastrectomy (TLDG). This minimally invasive procedure reduces postoperative pain, complications, and recovery period, in addition to enhancing the perioperative experience [8]. Nonetheless, TLDG must be performed by qualified and skilled surgeons to ensure a successful operation [9].

The R-Y and Billroth II with Braun (BII + B) reconstructions remain controversial as anti-biliary reflux procedures after distal gastrectomy. Both procedures have been compared in the literature, but most studies have emphasized short-term efficacy [10, 11]. Thus, this study compared the short- and long-term efficacy and QoL between R-Y and BII + B reconstructions.

## Materials and methods

### Participants

**Fig. 1:**
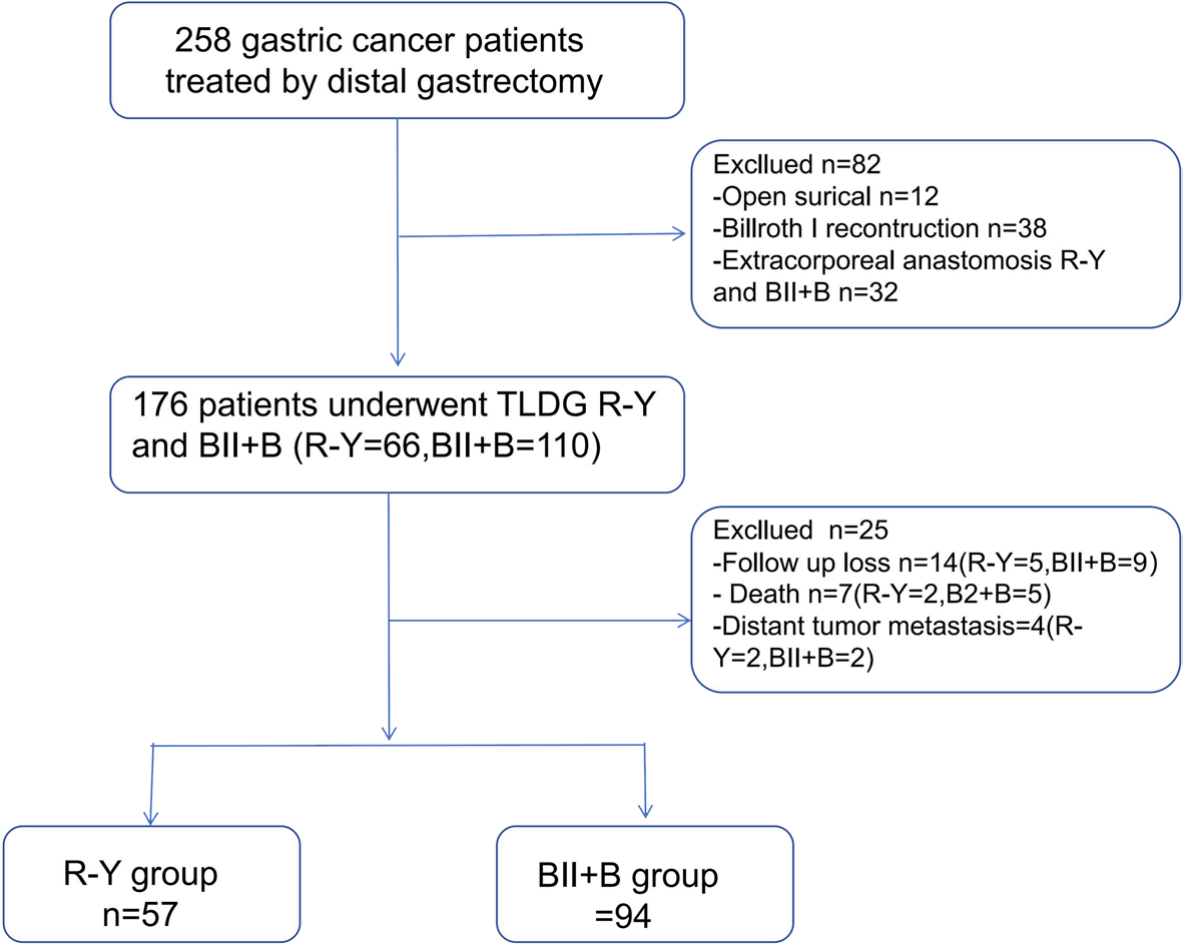
Study flow chart

This retrospective study was conducted at the Gastrointestinal Surgery Department of the Second Affiliated Hospital of Fujian Medical University, spanning from January 2016 to December 2019. Data were collected from gastric cancer patients who underwent distal gastrectomy by a single surgical team. Patients who underwent open surgery, the Billroth I procedure, or extracorporeal anastomosis involving R-Y and BII+ B procedures were excluded from the study. In total, 176 patients underwent TLDG utilizing the R-Y and BII + B procedures (R-Y = 66, BII + B = 110). Inclusion criteria encompassed patients who underwent TLDG with R-Y and BII + B procedures and received postoperative pathological diagnoses falling within stages I to III. Exclusion criteria included combined resection, intraoperative conversion to open surgery, loss to follow-up, death, or distant tumor metastasis. The final analysis comprised 151 patients, categorized into the R-Y group (*n* = 57), and the BII + B group (*n* = 94) (refer to Fig. 1). The preference for the reconstructive method gradually shifted toward the R-Y procedure in 2019 due to severe bile reflux observed following BII + B procedure.

### Surgical procedure

Gastrointestinal (GI) reconstruction after D2 lymph node dissection and distal gastrectomy.

#### Roux-en-Y reconstruction

**Fig. 2:**
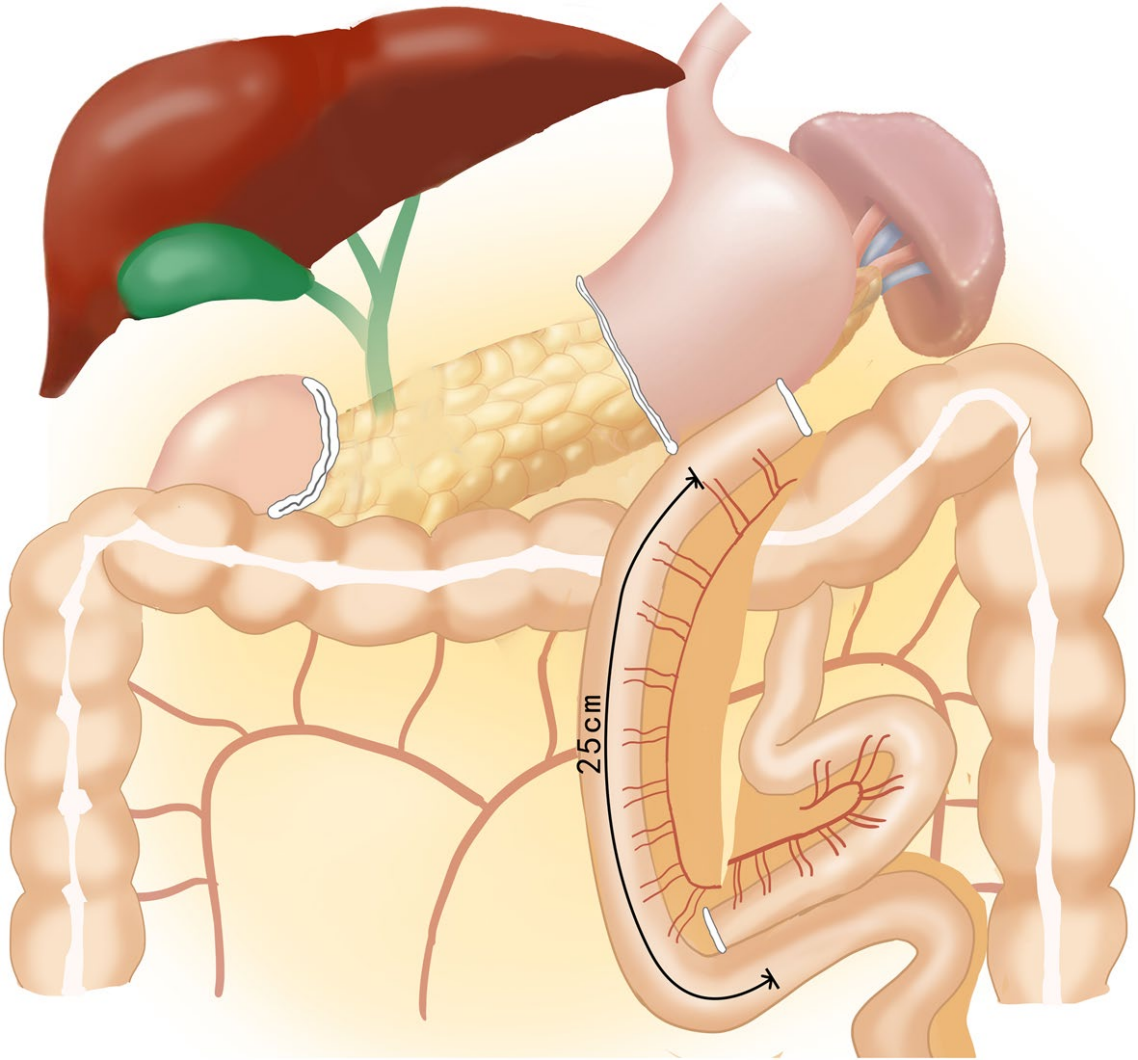
Roux-en-Y reconstruction

The jejunum was dissected 25 cm from the Treitz ligament. We performed a gastric-jejunal side-to-side anastomosis using a linear stapler and closed the common opening with manual sutures. Afterwards, a lateral jejuno-jejunal anastomosis was created 25 cm away from the gastrojejunal anastomosis (refer to Fig. 2).

#### BillrothII with Braun

**Fig. 3:**
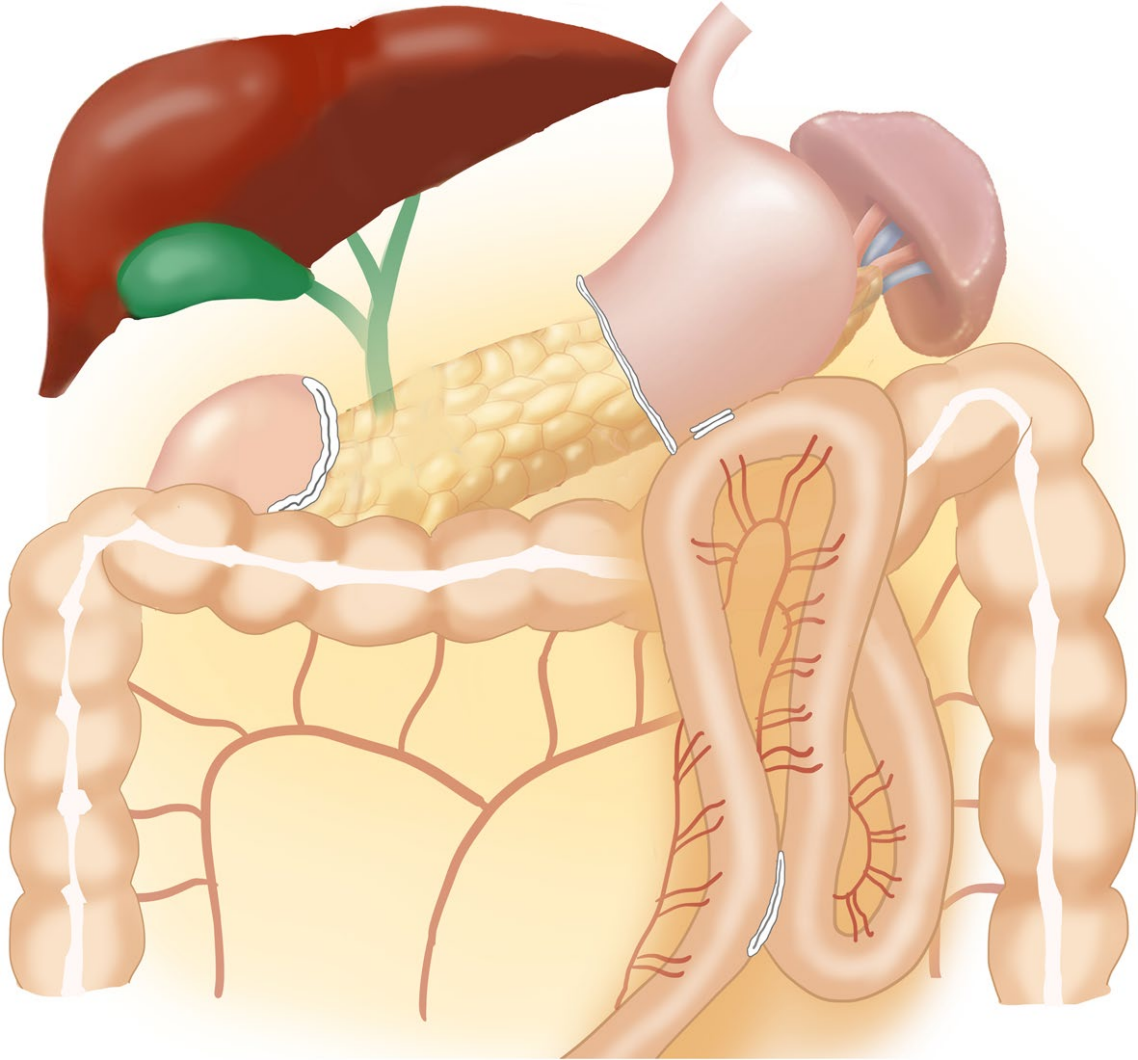
BillrothII with Braun reconstruction

A side-to-side gastrojejunal anastomosis was conducted 25 cm from the Treitz ligament, and the common gastrojejunal opening was closed using hand suturing. Additionally, a side-to-side jejuno-jejunal anastomosis was carried out 15 cm distal to the gastrojejunal anastomosis (refer to Fig. 3).

### Observation indexes

#### Clinicopathological data and perioperative variables

Clinicopathological data:sex, age, BMI (body mass index), tumor location, tumor size, differentiation degree, neoadjuvant therapy, TNM stage, ASA score, and follow-up times. The tumor TNM staging was determined according to the 8th edition of the American Joint Committee on Cancer (AJCC) [12]. ASA score was assessed based on the American Society of Anesthesiologists Classification [13].

Perioperative variables were blood loss, anastomotic time, operation time, time to initial exhaust, time to initial liquid food, time to the removal of the abdominal drain, and postoperative hospital days.

#### Early complications

Obstruction, anastomotic bleeding, delayed gastric emptying, anastomosis leakage, anastomotic stricture, intra-abdominal bleeding, intra-abdominal abscess, pancreatic fistula, chylous leakage, wound morbidity, Roux stasis syndrome (RSS), and pneumonia. RSS is defined as follows: (1) postoperative symptoms of induced nausea, vomiting, and abdominal distension; (2) perfect imaging, endoscopy to exclude mechanical obstruction (anastomotic stricture), and imaging suggestive of intestinal obstruction are not included in RSS [14]. According to the Clavien-Dindo classification, early complications were graded [15].

#### Late complications

Dumping syndrome, anastomotic ulcer, anastomotic stricture, anastomotic ileus, and intra-abdominal hernia. Dumping syndrome is defined as palpitations, hot flashes, excessive sweating, and hypoglycemia after eating.

#### Endoscopic findings

Endoscopic results at 1 and 3 years postoperation. Evaluation of postoperative residue, gastritis, and bile reflux was performed according to the Kubo M endoscopic grading method [16]. Reflux esophagitis was assessed according to the Los Angeles Grading System [17].

#### Nutrition

Body mass index (BMI), hemoglobin, protein, and albumin to the preoperative ratio in the 3rd year of post-surgery.

#### Quality of life assessment (3rd year post-surgery)

Quality of life in the third year post-surgery was assessed using the Quality of Life Questionnaire-Core 30 (QLQ-C30) and the Stomach Cancer Specific Modular Scale (QLQ-STO22), both developed by the European Organization for Research and Cancer Treatment (EORTC). These evaluations were carried out through outpatient follow-ups or telephone interviews. The QLQ-C30 questionnaire, comprising 30 items, encompasses five functional scales (physical, role, cognitive, emotional, and social), three symptom scales (fatigue, pain, nausea, and vomiting), six individual items, and a Global Health Status and Quality of Life scale. On the other hand, the EORTC QLQ-STO22 includes 22 items distributed across five symptom scales (dysphagia, epigastric pain, reflux, eating restrictions, anxiety) and four individual items [18].

All scale scores were linearly transformed to 0–100 scores. Higher scores on the global health status and QoL scale and the five functional scales of the QLQ-C30 indicate a better quality of life, while elevated scores on the three symptom scales and six individual items suggest a poorer quality of life. In the STO22, higher scores indicate greater symptom severity, reflecting a lower quality of life [19].

### Statistical analysis

Statistical analysis was conducted using SPSS 26.0 software in this study. Data with a normal distribution are presented as the mean ± standard deviation, and the *t*-test was employed for group comparisons. Skewed data are described as medians with 25th and 75th percentiles, and the Mann–Whitney *U* test was used for group comparisons. Categorical data were analyzed using the chi-square test or Fisher’s exact test. Rank data were compared using the nonparametric rank-sum test. Statistical significance was set at *p* < 0.05.

## Results

### Clinicopathological data and perioperative variables

Clinicopathological data from 151 patients, including sex, age, BMI, tumor location, tumor size, differentiation degree, neoadjuvant therapy, TNM stage, and ASA score, were collected for this study. These parameters were comparable between the R-Y and BII + B groups (*p* > 0.05). The R-Y group had a median follow-up time of 45 months (range 36–66), while the BII + B group had a median follow-up time of 59 months (range 44–72) (refer to Table 1).

**Table 1:** Clinicopathological data and perioperative variables

	R-Y (*n* = 57)	BII + B (*n* = 94)	*p* value
Clinicopathological data			
Sex			0.299
Male	36 (63.2)	67 (71.3)	
Female	21 (36.8)	27 (28.7)	
Age (years)	61.72 ± 11.08	60.29 ± 10.21	0.42
BMI (kg/m^2^)	22.25 ± 2.95	21.53 ± 2.82	0.143
Location of tumor			0.511
Middle third	18 (31.6)	25 (26.6)	
Lower third	39 (68.4)	69 (73.4)	
Tumor size (diameter, cm)	3.70 ± 1.90	3.63 ± 1.85	0.819
Differentiation			0.415
Low	30 (52.6)	60 (63.8)	
Medium	21 (36.8)	18 (19.1)	
High	6 (10.5)	16 (17.0)	
Neoadjuvant chemotherapy	6 (10.5)	13 (13.8)	0.553
pTNM-stage			0.351
I	29 (50.9)	38 (40.4)	
II	7 (12.3)	18 (19.1)	
III	21(36.8)	38 (40.4)	
ASA			0.865
I–II	49 (86.0)	80 (85.1)	
III	8 (14.0)	14 (14.9)	
Follow-up times: months	45 (36–66)	59 (44–72)	
Operative and postoperative conditions			
Blood loss(ml)	54.82 ± 21.67	50.41 ± 21.54	0.226
Anastomotic time (min)	36.96 ± 2.43	27.97 ± 3.74	0.001
Operative time (min)	194.65 ± 21.52	183.88 ± 18.02	< 0.001
Time to first exhaust (days)	2.0 (2.0; 3.0)	2.0 (3.0; 3.0)	0.125
Time to first liquid diet (days)	3.0 (3.0; 4.0)	3.0 (3.0; 4.0)	0.101
Time to remove abdominal drain (days)	8.0 (7.0; 9.0)	8.0 (7.0; 9.0)	0.199
Postoperative hospital days (days)	9.0 (8.0; 10.0)	9.5 (8.0; 10.0)	0.065

In terms of perioperative variables, no significant difference in blood loss was observed between the groups. However, there were statistically significant disparities in operative time (R-Y 194.65 ± 21.52 vs. BII + B 183.88 ± 18.02) and anastomotic time (R-Y 36.96 ± 2.43 vs. BII + B 27.97 ± 3.74) between the R-Y and BII + B groups (*p* < 0.05). Meanwhile, there were no significant differences between the postoperative conditions of both groups in terms of time to initially exhaust, time to initial liquid food, time to remove the abdominal drain, and postoperative hospital days (*p* > 0.05) (refer to Table 1).

### Early complications

The early complication rate was 10.5% in the R-Y group and 16.0% in the BII + B group. No significant difference was found between the two groups regarding the grade of complications (*p* > 0.05) (refer to Table 2).

**Table 2:** Early complications

	R-Y (n = 57)	BII + B (*n* = 94)	*p* value
Early complication	6 (10.5)	15 (16.0)	0.35
Intestinal obstruction	1 (1.8)	2 (2.1)	
Delayed gastric emptying	0	2 (2.1)	
Anastomotic bleeding	1 (1.8)	1 (1.1)	
Anastomosis leakage	0	2 (2.1)	
Anastomotic stricture	0	0	
Intra-abdominal bleeding	0	1	
Intra-abdominal abscess	1 (1.8)	2 (2.1)	
Pancreatic fistula	1 (1.8)	0	
Chylous leakage	0	1 (1.1)	
Wound morbidity	0	1 (1.1)	
RSS	1 (1.8)	0	
Pneumonia	1 (1.8)	4 (4.3)	
Clavien‒Dindo			0.368
I–II	3 (5.3)	9 (9.6)	
III	3 (5.3)	6 (6.4)	

### Late complications

Late complications occurred in 10.5% of the R-Y group and 12.8% of the BII + B group, but the difference between the two groups was not statistically significant (*p* = 0.931) (refer to Table 3).

**Table 3:** Late complications

	R-Y (*n* = 57)	BII + B (*n* = 94)	*p* value
Late complications	6 (10.5)	12 (12.8)	0.681
Dumping syndrome	5 (8.8)	7 (7.4)	
Anastomosis ulcer	0	1 (1.1)	
Anastomosis stricture	0	0	
Adhesive ileus	1(1.8)	2 (2.1)	
Internal hernia	0	2 (2.1)	

### Endoscopy findings

**Fig. 4:**
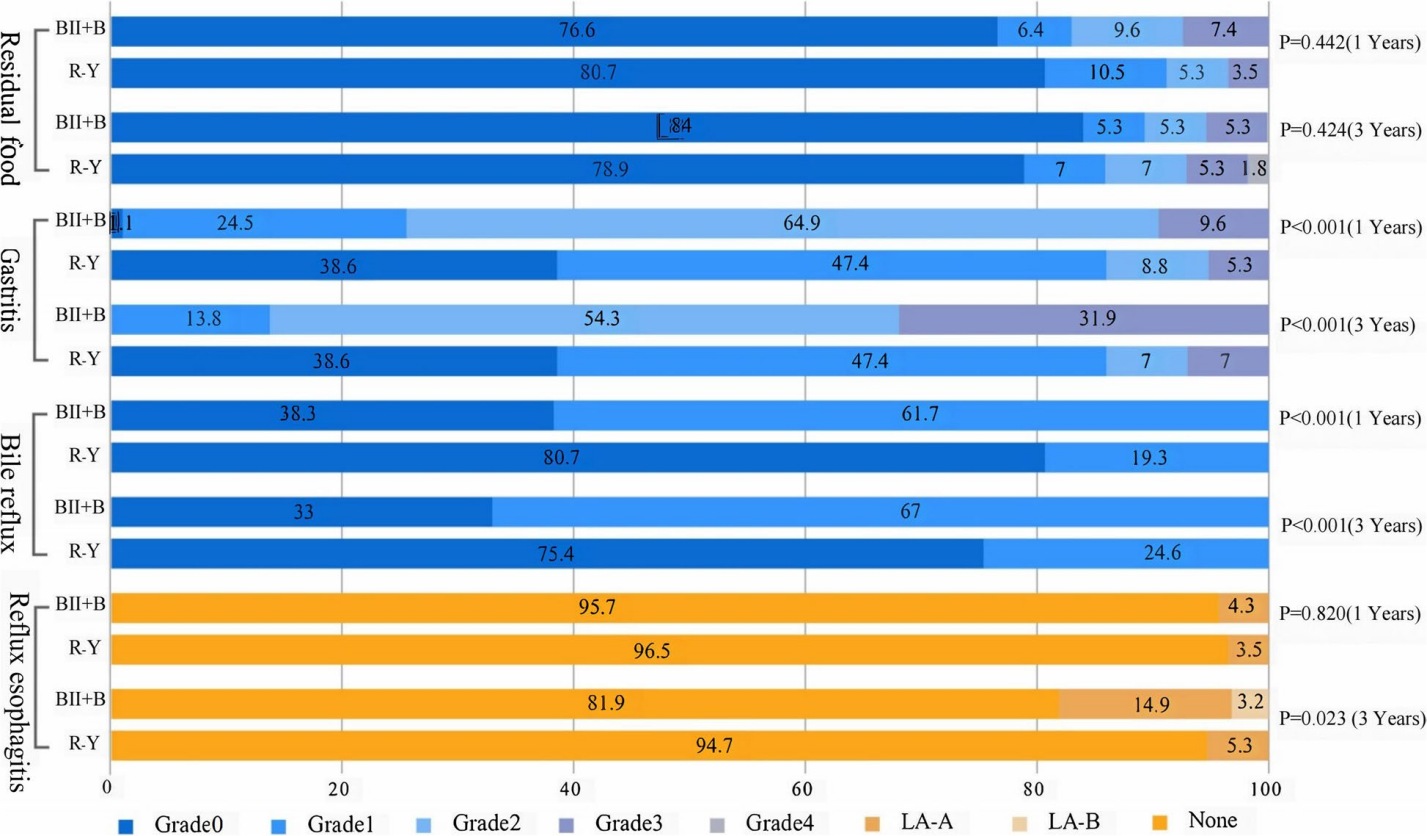
Comparison of endoscopic findings in the remnant stomach and reflux esophagitis at 1 and 3 years post-operation. Residue, gastritis, and bile reflux were graded using the RGB score system. Reflux esophagitis was classified according to the Los Angeles (LA) classification. The *x*-axis represents the percentage

There was no significant difference between both groups in terms of the endoscopic findings of food residues at years 1 and 3 (*p* > 0.05). Conversely, the occurrence of bile reflux and residual gastritis at years 1 and 3 was lower in the R-Y group than in the BII + B group (*p* < 0.001). Regarding reflux esophagitis, there was no significant difference between the groups in year 1, but in year 3, the R-Y group had a lower incidence compared to the BII + B group (*p* = 0.023) (refer to Fig. 4).

### Nutrition

The differences in BMI, hemoglobin, protein, and albumin to preoperative ratio at 3 years post-surgery were not statistically significant (*p* > 0.05) (refer to Table 4).

**Table 4:** Nutritional indicators to the preoperative ratio in the 3rd year after surgery

	R-Y (*n* = 57)	BII + B (*n* = 94)	*p* value
BMI (%)	97.66 ± 5.18	97.83 ± 7.45	0.873
Hemoglobin(%)	105.94 ± 19.53	107.32 ± 22.82	0.704
Protein (%)	106.53 ± 12.47	106.62 ± 16.69	0.97
Albumin (%)	103.32 ± 14.98	107.00 ± 19.51	0.224
Cholesterol (%)	96.70 ± 20.47	100.22 ± 32.03	0.423

*BMI* body mass index

### Quality of life assessment (3rd year post-surgery)

The differences between QLQ-C30 scores in all items were not found to be statistically significant (*p* > 0.05) (refer to Table 5). However, on the QLQ-STO22 scale, the R-Y group had a lower epigastric pain score than the BII + B group (0 [0,0] vs. 0 [0, 11.11]), although the difference was not statistically significant (*p* = 0.534). Additionally, the reflux score was significantly lower in the R-Y group than in the BII + B group (0 [0, 0] vs. 5.56 [0, 11.11]) (*p* = 0.003). The remaining items in the QLQ-STO22 did not show significant differences (*p* > 0.05) (refer to Table 6).

**Table 5:** QLQ-C30 scores at year 3 postsurgery

Items	R-Y (*n* = 57)	BII + B (*n* = 94)	*p* value
Global health status and QOL	83.33 (66.67, 83.33)	83.33 (66.67, 83.33)	0.419
Physical functioning	100 (86.67, 100)	100 (93.33, 100)	0.335
Role functioning	100 (100, 100)	100 (100, 100)	0.403
Cognitive functioning	100 (100, 100)	100 (100, 100)	0.707
Emotional functioning	100 (100, 100)	100 (100, 100)	0.135
Social functioning	100 (100, 100)	100 (100, 100)	0.449
Fatigue	16.67 (0, 16.67)	16.67 (12.50, 16.67)	0.622
Nausea and vomiting	0 (0, 0)	0 (0, 0)	0.816
Pain	0 (0, 0)	0 (0, 0)	0.726
Dyspnea	0 (0, 0)	0 (0, 0)	0.269
Insomnia	0 (0, 0)	0 (0, 0)	0.315
Appetite loss	0 (0, 0)	0 (0, 0)	0.505
Constipation	0 (0, 0)	0 (0, 0)	0.834
Diarrhea	0 (0, 0)	0 (0, 0)	0.492
Financial difficulties	0 (0, 0)	0 (0, 0)	0.42

**Table 6:** QLQ-STO22 score at year 3 postsurgery

Items	R-Y (*n* = 57)	BII + B (*n* = 94)	*p* value
Dysphagia	0 (0, 0)	0 (0, 0)	0.602
Epigastric pain	0 (0, 0)	0 (0, 11.11)	0.534
Reflux symptoms	0 (0, 0)	5.56 (0, 11.11)	0.003
Eating restriction	0 (0, 0)	0 (0, 0)	0.123
Anxiety	0 (0, 0)	0 (0, 0)	0.143
Having a dry mouth	0 (0, 0)	0 (0, 0)	0.199
Taste	0 (0, 0)	0 (0, 0)	0.436
Body image	0 (0, 0)	0 (0, 0)	0.586
Hair loss	0 (0, 0)	0 (0, 0)	0.269

## Discussion

Currently, TLDG has become a hot topic among surgeons. Ikeda O et al. [20] demonstrated that TLDG is associated with less invasiveness, reduced blood loss, smaller incisions, and a lower incidence of postoperative infections compared to LDG. In addition, Kanaya S et al. [21] reported the application of delta anastomosis, which facilitates the Billroth I procedure in TLDG. Although the reconstruction process of the R-Y is more complex, surgeons have made modifications to make it equally easy and safe in TLDG. Takaori K et al. [22] described the use of a linear stapler in isoperistaltic anastomosis R-Y reconstruction for TLDG. Okuno K et al. [23] proposed β-type R-Y reconstruction, which avoids the need for laparoscopic suturing.

Ideally, GI reconstruction after LDG should preserve residual gastric function, minimize long-term complications, and enhance the postoperative quality of life over the long term. The typical reconstruction involves Billroth I, Billroth II, and R-Y procedures. Billroth I procedure has the advantage of food passage in the duodenal pathway, but the risk of anastomotic fistula may be higher when the residual stomach is small; thus, this method is suitable for early-stage gastric patients [24]. In China, progressive gastric cancer is more common, and as a result, Billroth II and R-Y reconstructions more frequently performed. Billroth II reconstruction has the advantage of low tension but increases the risk of bile reflux gastritis and reflux esophagitis [25]. As a result, Braun made an anastomosis between the efferent and afferent loop at 10–15 cm from the gastrojejunal anastomosis based on Billroth II in 1892, effectively diverting partial bile and pancreatic fluid [26]. Nevertheless, this method has limitations, where studies still detected bile reflux and residual gastritis postsurgery [27]. In contrast, the R-Y method has the advantage of low-tension anastomosis. The long R-Y limb between the gastrojejunal and jejunojejunal anastomosis is beneficial for anti-bile reflux [4]. Nevertheless, previous studies indicated that RSS is often associated with nausea and vomiting post-surgery, possibly due to the disruption of intestinal electrophysiological continuity, thus affecting the patient’s QoL [28].

Surgical safety is a priority for surgeons when deciding the best reconstruction method. This study’s findings suggested that the R-Y group had significantly longer operative and anastomosis times compared to the BII + B group. Meanwhile, there was no significant difference in blood loss or postoperative recovery between the groups, which was consistent with studies by Chi F and Cui LH [11, 29]. R-Y procedure mesentery separation and jejunum dissection did not increase the risk of bleeding but prolonged the operative time. In addition, Yalikun A reported no significant difference in early postoperative complications between the R-Y (14.7%) and BII + B (7.5%) groups, which is similar to Shishegar A’s findings [30, 31]. In the present study, no significant differences were observed in the incidence of early complications (R-Y 10.5 vs. BII + B 16.0%) or late complications (R-Y 10.5 vs. BII + B 12.8%) between the two groups. Therefore, the safety of both procedures was comparable for TLDG.

RSS symptoms include stomach discomfort, bloating, and vomiting and are caused by delayed emptying in nonmechanical obstruction after the R-Y procedure, with an incidence rate of 10 to 30% [32]. The mechanism may be related to intestinal interruption, resulting in slow waves emitted by the “Y” limb that caused the limb to move retrogradely toward gastric motility [28].

However, the advancement of anastomotic devices and the modification of the traditional R-Y reconstruction by some surgeons in recent studies have significantly decreased the incidence of RSS. Gustavsson S demonstrated that an excessively long R-Y limb is a risk factor for RSS [14]. Motoyama K and An JY controlled the length of the “R-Y limb” to 25–30 cm, and none experienced RSS postoperatively [33, 34]. Additionally, our study utilized isoperistaltic R-Y reconstruction, which aligns more closely with human physiology and facilitates food emptying compared to antiperistaltic anastomosis [35]. Only one patient experienced RSS (1.8%), which was significantly lower than that in previous reports. Therefore, it was postulated that the 25-cm R-Y limb and the isoperistaltic gastrojejunal anastomosis potentially reduced RSS incidence.

Alkaline reflux gastritis caused by bile reflux is a significant factor that impacts the patient’s postoperative life [36]. Refluxed bile damage to the residual gastric mucosa results in residual gastritis, reflux esophagitis, and an increased risk of esophageal and residual gastric cancer [37, 38]. However, the effectiveness of the R-Y and BII + B procedures in preventing bile reflux remains controversial. Lee MS and Park JY reported that endoscopic findings a year after surgery exhibited significantly higher bile reflux and a higher degree of residual gastritis in the BII + B group than in the R-Y group [39, 40]. In contrast, Yalikun A indicated that a modified BII + B reconstruction with prolonged afferent and efferent loops had postoperative bile reflux and residual gastritis comparable to R-Y reconstruction but may increase the risk of afferent limb torsion and internal hernia [40].

In the current study, the endoscopic findings at years 1 and 3 post-surgery revealed significantly lower levels of residual gastritis and occurrences of bile reflux in the R-Y group compared to the BII + B group. The superiority of R-Y reconstruction in terms of anti-reflux effects can be attributed to several factors. First, the Billroth II gastrojejunal anastomosis lacks anti-reflux properties, allowing bile from the afferent loop to flow into the residual stomach along with intestinal peristalsis. Second, even with the Braun procedure diverting bile, some bile may still reflux to the residual stomach since the Braun anastomosis is positioned only 10–15 cm away from the gastrojejunostomy. In contrast, R-Y reconstruction anastomoses the proximal jejunum with the distal jejunum, preventing the direct flow of bile into the remaining stomach. Finally, the long and steep limb between the gastrojejunal and jejunojejunal anastomosis in R-Y reconstruction makes bile reflux into the residual stomach difficult.

Chung JH’s endoscopic findings at 6–32 months demonstrated that the proportion of grade 2 and 3 residual gastritis increased from 73.0 to 86.1% in the BII + B group, and the incidence of reflux esophagitis rose from 1 to 15.7% [41]. Similarly, Choi C’s endoscopic findings at year 2 postoperatively showed increased bile reflux and an aggravated degree of residual gastritis in the BII + B group compared to year 1, with significantly higher reflux esophagitis compared to the R-Y group [42]. In the current study, the proportion of grade 2 and 3 residual gastritis increased in the BII + B group at year 3 postoperatively by 86.2% compared with 74.5% at year 1. Conversely, there was no significant change in the R-Y group. Furthermore, there was no significant difference in the degree of reflux esophagitis between the two groups in year 1, but in year 3, the occurrence was significantly lower in the R-Y group than in the BII + B group (*p* = 0.023). These findings suggest that the degree of residual gastritis and reflux esophagitis due to bile reflux may worsen over time, indicating that the R-Y group has a better long-term prognosis.

Moreover, when the residual stomach is smaller, the distance between the gastrojejunal anastomosis and the cardia is reduced. This shortened distance increases the likelihood of bile damaging the esophageal mucosa, leading to reflux esophagitis. Therefore, the R-Y method is advantageous when the patient’s residual stomach is small. Additionally, the two groups had no significant differences in BMI, hemoglobin, protein, or albumin to the preoperative ratio at year 3 post-surgery. Likewise, Lee MS and Park JY also reported similar results [39, 40].

Gastric surgery alters digestive structure and function, leading to post-gastrectomy symptoms such as restricted eating, appetite loss, and reflux, significantly impacting long-term quality of life. Lee MS’s study suggested that R-Y procedure reduces bile reflux, but at 6 and 12 months post-surgery, the Gastrointestinal Quality of Life Index scores, including symptoms, physical, emotional, and social functions, showed no significant difference compared to Billroth I and BII + B methods [39]. Cui L compared R-Y and BII + B groups at 6 months post-surgery and found no significant differences in gastrointestinal symptom scores [29]. However, Chan DC et al. indicated that R-Y reconstruction can reduce bile reflux-related abdominal pain and heartburn, enhancing quality of life after surgery [43].

The combined use of EORTC QLQ-30 and STO22 questionnaires allows a comprehensive evaluation of post-gastrectomy Qol from physical, and mental health, and specific gastric cancer-related symptoms. Smolskas E’s study revealed no significant differences in QLQ-30 scores 1 year after surgery among the Billroth I, Billroth II, and R-Y groups. However, specific gastric cancer symptoms were not assessed using STO22 [44]. Yang K’s research demonstrated that R-Y reconstruction reduces reflux, leading to a better long-term quality of life compared to Billroth I [45]. This study’s results indicate that 3 years after surgery, R-Y and BII + B groups show no significant differences in the QLQ-30 scales. However, in the STO22 scale, the R-Y group had lower scores for epigastric pain and reflux symptoms compared to the BII_B group, indicating a better postoperative quality of life.

However, this study has limitations. It is a single-center retrospective study with small sample size. Preoperative QOL was not assessed, making it impossible to establish a baseline. Additionally, postoperative QoL was evaluated at a single time point, which does not capture the dynamic changes in QoL over time. Future research will involve large-scale, multicenter prospective studies to address these limitations. Furthermore, we have collated findings from prior studies focusing on surgical and endoscopic outcomes, nutrition, and quality of life, to offer a comparative perspective with the results of our current research (refer to Table 7).

**Table 7:** Results of studies reviewed

Author, year, country	Study design	Study period	Procedure	*n*	Primary Surgical outcome	Endoscopic findings	Nutrition	Quality of life
In Choi C, 2016, Korea [42]	Retrospective study	2010/4–2012/8	R-Y	26	1. R-Y had a longer operation time 2. The incidence of postoperative complications did not differ statistically between the groups	1. At 1-year and 2-year post-surgery, the BII group experienced higher rates of bile reflux and residual gastritis 2. By the 2-year mark, reflux esophagitis was significantly higher in the BII group compared to R-Y	None	None
			BII + B	40				
Lee MS, 2011, Korea[39]	Prospective study	2006/3–2007/8	BI	49	1. The B-I group had significantly shorter operation times 2. Postoperative complications did not differ significantly among the three groups	At 1-year post-surgery, the R-Y group exhibited significantly lower bile reflux and residual gastritis compared to the other two groups.	At 1-year post-surgery, there was no significant difference in nutritional indicators (BMI, hemoglobin, albumin) among the three groups	At 6 and 12 months after surgery, there was no significant difference in the total GIQLI score and the subscale scores (symptoms, physical, emotional, and social functioning) among the three groups
			R-Y	47				
			BII + B	52				
Shishegar A, 2022, Iran [31]	Prospective study	2018/1–2020/1	R-Y	42	1. Operative time and blood loss were higher in the R-Y group compared to the BII group 2. There was no significant difference in early postoperative complications and long-term complications within 1 year between the two groups	At 6 months postoperatively, there was no difference observed in the occurrence of residual gastritis and reflux esophagitis between the two groups	None	None
			BII + B	42				
Chi F, 2021, China [11]	Retrospective study	2019/1–2020/7	R-Y	51	1. R-Y had a longer operation and anastomosis times compared to BII 2. Surgical safety and postoperative recovery were comparable between the two groups	None	None	None
			BII + B	54				
Yalikun A, 2022, China [30]	Retrospective study	2016/1–2019/12	R-Y	102	1. The modified BII procedure had shorter anastomosis and operative times compared to R-Y 2. There was no significant difference in the incidence and severity of postoperative complications between the two groups	At 6 months and 1 year post-surgery, the modified BII procedure showed no significant difference in the incidence of bile reflux and residual gastritis compared to R-Y	None	None
			BII + B	145				
Cui L, 2017, Korea [29]	Retrospective study	2023/1–2015/12	R-Y	30	1. R-Y had a longer operation time 2. The two groups showed no significant differences in blood loss, time to first flatus, and postoperative complications	6 months after surgery, there was no significant difference in the incidence of bile reflux or residual gastritis between the two groups	6 months after surgery, there was no significant difference in body mass index (BMI) variations between the two groups	6 months after surgery, there was no significant difference in the occurrence of gastrointestinal symptoms (diarrhea, constipation, dyspepsia, flatulence, and reflux) between the two groups
			BII + B	26				
Park JY, 2014, Korea [40]	Retrospective study	2005/3–2013/10	R-Y	55	1. The operating time was longest in the RY group and shortest in the uncut RY group 2. Blood loss was the lowest in the uncut RY group 3. Postoperative complications and recovery did not differ statistically among the three groups	1 year after surgery, the incidence of bile reflux and residual gastritis was lower in the R-Y and Uncut R-Y groups	1 year after surgery, there was no significant difference in nutritional indicators (BMI, hemoglobin, albumin) among the three groups	None
			BII + B	76				
			BI	39				
			Uncut R-Y	41				
Chan DC, 2007, China [43]	Retrospective study	2002/1–2005/1	R-Y	19	1. RY had less postoperative output from the nasogastric tube 2. Postoperative complications are not statistically different among groups	1. After 1 year, biliary scintigraphy showed less R-Y enterogastric reflux than B2 and BII procedures 2. R-Y had lower bile reflux (15.8%) compared to BII (75.9%) and BII (83.3%), with milder gastritis 3. R-Y group had less Helicobacter pylori infection than others	None	1 year after surgery, symptoms of distal gastrectomy (epigastric pain, heartburn, biliary vomiting, postprandial bloating, and nausea) were evaluated, and the R-Y group experienced the mildest symptoms
			BII	29				
			BII + B	12				

*R-Y* Roux-en-Y reconstruction, *BI* BillirothI reconstruction, *BII* Billroth II reconstruction, *BII* + *B* BillrothII with Braun reconstruction

## Conclusion

The anastomotic time of R-Y reconstruction in TLDG is longer than that of BII + B reconstruction. However, there is no increased risk of bleeding associated with R-Y reconstruction. Postoperative recovery and surgical safety were comparable in both groups. Nonetheless, R-Y reconstruction exhibits a superior anti-biliary reflux effect, reducing residual gastritis and reflux esophagitis in comparison to BII + B reconstruction. Furthermore, R-Y reconstruction diminishes postoperative reflux symptoms and enhances the QoL. Both procedures demonstrate safety and feasibility, but the R-Y surpasses the BII + B procedure for TLDG. Future prospective studies should be conducted involving multiple centers and a larger sample size to further validate the results of this study.

## Data Availability

The original anonymous dataset is available on request from the corresponding author at e5179518qihe9323@163.com.

## Abbreviations

QoL: Quality of Life
R-Y: Roux-en-Y
LDG: Laparoscopic distal gastrectomy
LADG: Laparoscopic-assisted distal gastrectomy
TLDG: Total laparoscopic distal gastrectomy
BII + B: Billroth II with Braun
BMI: Body mass index
RSS: Roux stasis syndrome
